# Insect Sex Determination Manipulated by Their Endosymbionts: Incidences, Mechanisms and Implications

**DOI:** 10.3390/insects3010161

**Published:** 2012-02-10

**Authors:** Daisuke Kageyama, Satoko Narita, Masaya Watanabe

**Affiliations:** Insect-Microbe Research Unit, National Institute of Agrobiological Sciences (NIAS), Owashi 1-2, Tsukuba, Ibaraki 305-8634, Japan; E-Mails: naritas@affrc.go.jp (S.N.); wtnbm1ab@affrc.go.jp (M.W.)

**Keywords:** endosymbiont, feminization, insect, male killing, *Wolbachia*, sex determination

## Abstract

The sex-determining systems of arthropods are surprisingly diverse. Some species have male or female heterogametic sex chromosomes while other species do not have sex chromosomes. Most species are diploids but some species, including wasps, ants, thrips and mites, are haplodiploids (n in males; 2n in females). Many of the sexual aberrations, such as sexual mosaics, sex-specific lethality and conversion of sexuality, can be explained by developmental defects including double fertilization of a binucleate egg, loss of a sex chromosome or perturbation of sex-determining gene expression, which occur accidentally or are induced by certain environmental conditions. However, recent studies have revealed that such sexual aberrations can be caused by various groups of vertically-transmitted endosymbiotic microbes such as bacteria of the genera *Wolbachia*, *Rickettsia*, *Arsenophonus*, *Spiroplasma* and *Cardinium*, as well as microsporidian protists. In this review, we first summarize the accumulated data on endosymbiont-induced sexual aberrations, and then discuss how such endosymbionts affect the developmental system of their hosts and what kinds of ecological and evolutionary effects these endosymbionts have on their host populations.

## 1. Introduction

In arthropods, the fundamental system of sex determination is generally considered to be genetic, and this is supported by the strict concordance in the dimorphism of sexual phenotypes and karyotypes (*i.e.*, sex chromosomes) and the rare occurrence of gynandromorphs (chimeric individuals having tissues with male and female genotypes). However, the sex determination and differentiation of arthropods can be perturbed by the endosymbionts or parasites they harbor. The most striking effects on sexual phenotype are induced by microbes that are transmitted from mothers to offspring (often called maternally transmitted or cytoplasmic parasites). Since males cannot transmit cytoplasmic parasites to their offspring, parasites in the male cytoplasm are essentially dead from the evolutionary viewpoint. To make up for these shortcomings, some parasites adopt a variety of tactical strategies such as killing of males, converting males into females or inducing parthenogenesis.

In this article, we briefly overview the diversity of the sex-determining mechanisms in insects, summarize the incidences of maternally transmitted microbes that affect or may affect sex determination, and discuss the possible mechanism of such phenomena. We also discuss the ecological and evolutionary effects that such microbes potentially have on their host populations.

## 2. Diversity and Common Features of Insect Sex Determination

**Figure 1:**
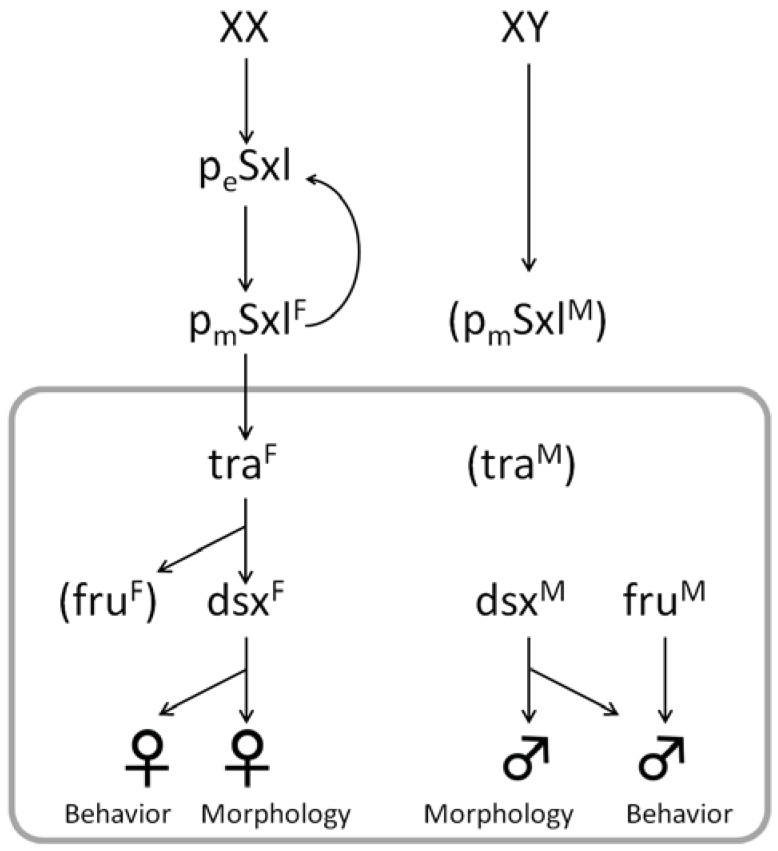
The sex determination cascade in *Drosophila melanogaster*. PeSxl indicates *Sxl* transcript from the early promoter. PmSxl indicates *Sxl* from the late promoter. The gray box shows the conserved part of the cascade. (Adapted from [7].)

In the majority of insects, sex is strictly determined according to the genotype. For example, many of the dipteran insects (flies and mosquitoes) have a male-heterogametic sex chromosome constitution (*i.e.*, XX: female; XY: male) while many of the lepidopteran insects (butterflies and moths) have a female-heterogametic chromosomal constitution (*i.e.*, ZZ: male; ZW: female). On the other hand, many of the hymenopteran insects (ants, bees and wasps) do not have sex chromosomes. Instead, they have a haplodiploid sex-determination system, in which fertilized diploid (2n) eggs become females and unfertilized haploid (n) eggs develop into males [1,2,3]. The molecular mechanisms underlying sex determination and differentiation in the model insect *Drosophila melanogaster* (Diptera; Drosophilidae) are well understood. At a very early embryonic stage, each cell determines its sex independently, and once determined, the sex of each cell is maintained during later development through a gene expression cascade consisting of *Sex-lethal* (*Sxl*), *transformer* (*tra*), *doublesex* (*dsx*) and other genes, in which sex-specific mRNA splicing plays an important role [4,5,6]. In the honeybee *Apis mellifera*, the *complementary sex determiner* (*csd*) gene affects sex through allelic combination, whereas the *feminizer* (*fem*) gene induces sex-specific splicing, producing a functional protein only in females. Comparisons between the sex-determination pathways of *A. mellifera*, *Ceratitis capitata* and *D. melanogaster* suggest that the *tra*/*dsx* pathway is conserved among insects and is likely to be ancestral [7] (Figure 1). Sex determination in a cell-autonomous manner is also believed to be widespread among insects on the basis that sexually mosaic individuals often occur in a diverse array of insects [8].

Similar to insects, some of the non-insect arthropods are also considered to have genetically based sex determination. However, they differ from insects because their sexual differentiation is deeply affected by sex hormones that are secreted by particular organs (e.g., the androgenic gland in crustaceans) and circulate in the body together with hemolymph.

## 3. Sexual Aberrations of Insects

In arthropods, sexually mosaic or gynandromorphic individuals are repeatedly observed in natural and laboratory populations of numerous species [9]. Most of them are considered to arise through accidental, very rare developmental defects such as double fertilization of a binucleate egg, loss of a sex chromosome or upregulation/downregulation of sex-determining genes.

On the other hand, some conditions can stably generate sexual aberrations in certain insects. In some mosquitoes of the genus *Aedes*, a high temperature condition can cause males to develop into morphological females. When the number of days of exposure to high temperature was lessened, various degrees of intersexes, *i.e.*, individuals having both male and female characters, appeared [10,11,12]. Crosses between geographical races of the gypsy moth *Lymantria dispar* also resulted in the appearance of intersexes [13,14,15]. In some crossing combinations, genetically male offspring (ZZ) showed mosaic color patterns in their wings, which is clearly distinguishable between sectors of male color and female color. In other combinations, genetically female offspring (ZW) showed homogeneous in their wing color, which is intermediate between male and female [14]. Sexual mosaics were also found in crosses between tetraploid females and diploid males in the psychid moth *Solenobia triquetrella* [16]. The resulting triploid offspring with two Z chromosomes and three sets of autosomes developed into sexual intergrades owing to the intermediate ratio (2:3) between the Z chromosomes and autosome sets, which is neither typically female (1:2) nor typically male (1:1). Although it was used to be considered that all the aberrations of sexuality were attributed to environmental disturbances or stochastic effects, recent investigations revealed that some of them can be caused by endosymbionts, another organism living inside the body.

## 4. Involvement of Endosymbiotic Microorganisms

It has been increasingly recognized that various forms of sex aberrations in arthropods can be caused by symbiotic microorganisms. It was first recognized that *Wolbachia pipientis* (belonging to Alphaproteobacteria) exerts various types of manipulation on the reproductive system of arthropods [17,18,19]. *Wolbachia*-induced reproductive manipulation can be classified into four major categories: feminization, male killing, parthenogenesis induction and cytoplasmic incompatibility. By using these manipulations, *Wolbachia* can spread among host populations at the expense of host fitness. Following the succession of discoveries regarding *Wolbachia*-induced host manipulations, several other bacteria such as *Cardinium*, *Spiroplasma* and *Arsenophonus*, which belong to distantly related taxonomic groups, were also found to manipulate host reproduction in similar ways to *Wolbachia* (Table 1 and Table 2; Table S1).

Similar to other symbiotic bacteria such as *Buchnera* [20] and *Wigglesworthia* [21], *Wolbachia* resides in the cytoplasm of the host cells and mainly relies on maternal inheritance within arthropod lineages. However, in contrast to mutualistic symbionts, phylogenetic analyses suggest that *Wolbachia* have moved horizontally between distantly related insect lineages multiple times possibly through host–parasite or predator–prey interactions [22,23,24].

**Table 1:** Endosymbionts that are capable of manipulating host sexuality and reproduction.

Endosymbiont				Hosts	Phenotype
Kingdom	Phylum	Class	Species		
Bacteria	Proteobacteria	α-Proteobacteria	*Wolbachia pipientis*	insects, crustaceans, arachnids, nematodes	CI, MK, PI, FM
			*Rickettsia* sp.	insects	MK, PI
		γ-Proteobacteria	*Arsenophonus nasoniae*	insects	MK
	Firmicutes	Mollicutes	*Spiroplasma poulsonii*	insects	MK
			*Spiroplasma ixodetis* relative	insects	MK
	Cytophaga-Flavobacterium-Bacteroides	Bacteroidetes	*Cardinium hertigii*	insects, arachnids, nematodes	CI, PI, FM
			Flavobacteria relative	insects	MK
Eukaryotes	Microsporidia	Dihaplophasea	*Octosporea effeminans*	crustaceans	FM
			*Thelohania herediteria*	crustaceans	FM
			*Nosema granulosis*	crustaceans	FM
			*Dictyocoela duebenum*	crustaceans	FM
			*Amblyospora* spp.	insects	MK
			*Parathelohania legeri*	insects	MK
			*Parathelohania obesa*	insects	MK
	Nematoda	Adenophorea	*Gasteromermis* sp.	insects	FM
Viruses	Unknown	Unknown	Unknown	crustaceans	MS
			Unknown (RNA virus)	insects	MK

Abbreviations: CI, cytoplasmic incompatibility; MK, male killing; PI, parthenogenesis induction; FM, feminization; MS, masculinization.

**Table 2:** A List of endosymbionts and their hosts, where feminization, male killing and parthenogenesis induction were described.

Endosymbiont	Class	Order	Species	Reference
**(a) Feminizing bacteria**				
*Wolbachia pipientis*	Insecta	Hemiptera	*Zyginidia pullula*	[28]
		Lepidoptera	*Eurema hecabe*	[27]
			*E. madarina*	[25,26,129]
	Malacostraca	Isopoda	*Armadillidium vulgare*	[130,131,132]
			*A. nasatum*	[132,133,134]
			*Chaetophiloscia elongata*	[135]
			*Porcellionides pruinosus*	[135,136]
			*Sphaeroma rugicauda*	[137]
*Cardinium hertigii*	Insecta	Hymenoptera	*Encarsia hispida*	[44]
	Arachnida	Trombidiformes	*Brevipalpus phoenicis*	[40]
			*B. californicus*	[42]
**(b) Feminizing microsporidia**				
*Octosporea effeminans*	Malacostraca	Amphipoda	*Gammarus duebeni*	[46]
*Thelohania herediteria*			*G. duebeni*	[138]
*Nosema granulosis*			*G. duebeni*	[48]
*Dictyocoela duebenum*			*G. duebeni*	[52]
**(c) Other feminizers**				
*Gasteromermis* sp.	Insecta	Ephemeroptera	*Baetis bicaudatus*	[53]
f factor (unknown)	Malacostraca	Isopoda	*Armadillidium vulgare*	[33]
**(d) Male-killing bacteria**				
*Wolbachia pipientis*	Insecta	Coleoptera	*Adalia bipunctata*	[139]
			*Tribolium madens*	[140]
		Diptera	*Drosophila bifasciata*	[141]
			*D. borealis*	[142]
			*D. innubila*	[143]
		Lepidoptera	*Acraea encedon*	[128]
			*A. encedana*	[144]
			*Hypolimnas bolina*	[69]
			*Ostrinia furnacalis*	[64]
			*O. orientalis*	[145]
			*O. scapulalis*	[63,146]
			*O. zaguliaevi*	[145]
	Arachnida	Pseudoscorpionida	*Cordylochernes scorpioides*	[147]
*Spiroplasma ixodetis *relatives	Insecta	Coleoptera	*Adalia bipunctata*	[148,149]
			*Anisosticta novemdecimpunctata*	[150]
			*Harmonia axyridis*	[151,152]
			*Menochilius sexmaculatus*	[153]
		Hemiptera	*Acyrthosiphon pisum*	[77,79]
		Lepidoptera	*Danaus chrysippus*	[154]
			*Ostrinia zaguliaevi*	[155]
*Spiroplasma poulsonii*		Diptera	*Drosophila nebulosa*	[156]
			*D. neocardini*	[157]
			*D. melanogaster*	[158]
			*D. ornatifrons*	[157]
			*D. paraguayensis*	[157]
			*D. willistoni*	[159]
*Rickettsia *spp.	Insecta	Coleoptera	*Adalia bipunctata*	[149,160,161]
			*A. decempunctata*	[162]
			*Brachys tesselatus*	[163]
			*Propylea japonica*	[164]
Flavobacteria	Insecta	Coleoptera	*Adonia variegate*	[165]
			*Coccinula sinensis*	[166,167]
			*Coleomegilla maculata*	[168]
*Arsenophonus nasoniae*	Insecta	Hymenoptera	*Nasonia vitripennis*	[169,170]
**(e) Male-killing microsporidia**				
*Parathelohania legeri*		Diptera	*Anopheles quadimaculatus*	[171]
*Parathelohania obesa*			*A. quadimaculatus*	[172]
*Amblyospora *spp.			*Aedes *spp.	[173,174]
*Amblyospora *spp.			*Culex *spp.	[174,175,176,177]
*Amblyospora *spp.			*Culiseta *spp.	[174]
**(f) Other male killers**				
Unknown virus	Insecta	Lepidoptera	*Homona magnanima*	[59,178]
			*Armadillidium vulgare*	[56]
			*Porcellio dilatatus*	[56]
			*P. laevis*	[56]
**(g) Parthenogenesis-inducing bacteria**				
*Wolbachia pipientis*	Insecta	Hymenoptera	*Aphytis diaspidis*	[179,180]
			*A. lignaensis*	[179,181]
			*Aponanagyrus diversicornis*	[91]
			*Asobara japonica*	[92]
			*Diplolepsis rosae*	[182]
			*Encarsia formosa*	[90]
			*Eretmocerus mundus*	[183]
			*Gronotoma micromorpha*	[184]
			*Muscidifurax uniraptor*	[185,186]
			*Telenomus nawai*	[187]
			*Trichogramma brevicapilliun*	[188]
			*T. chilonis*	[189]
			*T. cordubensis*	[188,189]
			*T. deion*	[93,188,189]
			*T. embryophagum*	[188,189]
			*T. evanescens*	[188,189]
			*T. kaykai*	[190]
			*T. oleae*	[132,189]
			*T. platneri*	[188,189]
			*T. pretiosum*	[93,188,189]
	Insecta	Thysanoptera	*Franklinothrips vespiformis*	[88]
	Arachnida	Trombidiformes	*Bryobia praetiosa*	[40]
			*Bryobia *sp.	[40]
*Cardinium hertigii*	Insecta	Hymenoptera	*Encarsia hispida*	[44]
			*E. pergandiella*	[191]
			*E. protransvena*	[191,192]
	Arachnida	Trombidiformes	*Brevipalpus phoenicis*	[43]
*Rickettsia *spp.	Insecta	Hymenoptera	*Neochrysocharis formosa*	[96,193]
			*Pnigalio soemius*	[194]

## 5. Endosymbiont-Induced Feminization: Examples Are Scarce but May Potentially Be More Common

For maternally transmitted microbes, males are an evolutionary dead end. To circumvent this problem, some microbes convert males into functional females so that they can be transmitted to subsequent generations. In insects, microbe-induced feminization has only been found in butterflies and leafhoppers, wherein *Wolbachia* is the causal agent of feminization. Among non-insects, woodlice, mites and shrimps are known to be feminized by *Wolbachia*, *Cardinium* and microsporidians (e.g., *Octosporea*), respectively (Table 2).

### 5.1. Feminization of Butterflies by *Wolbachia*

**Figure 2:**
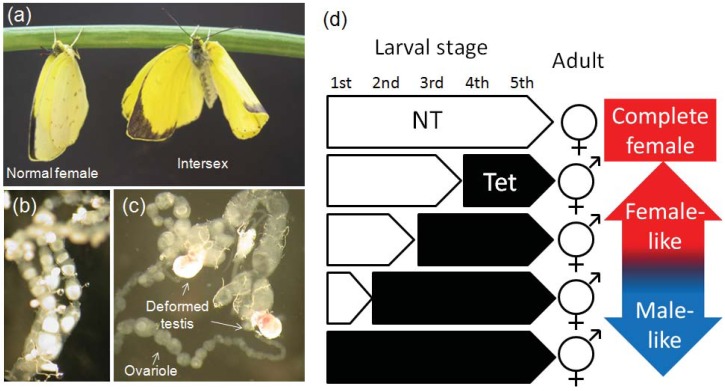
Effects of the antibiotic on the sexual phenotype of the butterfly *Eurema mandarina* infected with two strains of *Wolbachia* [26]. (**a**) A normal female and an intersex generated by antibiotic treatment. The intersex has both male and female features in external morphology. (**b**) Ovarioles of normal females. (**c**) The reproductive organs of the intersex. Both male-specific organs (testes) and female-specific organs (ovarioles) occur in an individual treated with tetracycline hydrochloride during larval stages. (**d**) Schematic illustration showing the effect of antibiotic treatment of larvae on adult sexual phenotype. The longer the treatment, the more male-like phenotype appears. NT: non-treated. Tet: tetracycline-treated.

In two islands located in the southern part of Japan (Tanegashima Island and Okinawa Island), some females of the butterfly *Eurema mandarina* (Lepidoptera; Pieridae) are known to have a chromosomal constitution of males (ZZ) instead of females (ZW). This incongruence between chromosomal and phenotypic sex can be explained by feminization of genetic males induced by *Wolbachia* [25,26]. Two distinct strains of *Wolbachia*, *w*CI and *w*Fem, have been found in *E. mandarina* inhabiting these islands. Females having male chromosomes (ZZ) are consistently infected with both *w*CI and *w*Fem, while females singly infected with *w*CI are true females (ZW). Despite having complete male chromosomes, ZZ females are morphologically and behaviorally completely female and fully fertile. Moreover, when such individuals are treated with tetracycline hydrochloride, a bacteriostatic antibiotic, during larval development, they develop as butterflies with intersexual morphology. Before carrying out this experiment, the appearance of intersexes in the treated generation was not expected because perturbation of sex determination was believed to occur during early embryogenesis when the sex-determining genes start to be expressed. However, the appearance of intersexes clearly shows that *Wolbachia* needs to be present during larval development for complete feminization. The fact that butterflies with more male-like characters were generated after longer antibiotic treatments shows that *Wolbachia* acts continuously during larval development [26] (Figure 2). The presence of *Wolbachia* during the embryonic stage seems to be necessary for complete feminization since antibiotic treatment during the whole larval stages also generated intersexes instead of complete males. We recently found that the splicing pattern of the sex-determining gene *doublesex* (*dsx*) changes according to the *Wolbachia* infection status (Narita *et al.*, in preparation). *Wolbachia*-induced feminization has also been found in *Eurema hecabe*, a sibling species of *E. mandarina*. The two *Wolbachia* strains found in feminized *E. hecabe* are indistinguishable from *w*CI and *w*Fem derived from *E. mandarina* based on multi-locus sequence typing [27].

### 5.2. Feminization of Leafhoppers by *Wolbachia*

A second case of *Wolbachia*-induced feminization in diploid insects has been found in the leafhopper *Zyginidia pullula* (Homoptera; Cicadellidae) [28]. In *Z. pullula*, females have two X chromosomes (2n = 8AA + XX) while males have only one X chromosome (2n = 8AA + X0). This is contrary to the situation of the *Eurema* butterflies wherein females have one Z chromosome and males have two Z chromosomes. When *Wolbachia*-infected *Z. pullula* females collected in northern Italy were mated with males, they produced exclusively female broods. Close inspection of these female broods revealed that about half of them had an intersexual morphology (*i.e.*, showing upper pygofer appendages, a typical male secondary sexual character), while the rest of the broods had the normal female phenotype. While the karyotypes of the normal females were XX, those with the upper pygofer appendages were X0 and were thus feminized males. Administration of tetracycline hydrochloride to *Wolbachia*-infected females (phenotypically normal female) resulted in a nearly 1:1 sex ratio in the subsequent generation [28]. The incongruence between the phenotypic sex and genomic sex implies the presence of epigenetic modification. Negri *et al.* [29] compared the DNA methylation patterns between intersexual and normal *Z. pullula* by performing a methylation-sensitive random PCR and found that females with the male upper pygofer appendages showed a female methylation pattern. On the other hand, some rare feminized males bore testes instead of ovaries. And these individuals showed a male methylation pattern. These findings suggest that *Wolbachia* induces feminization by disrupting male imprinting.

### 5.3. Feminization of Woodlice by *Wolbachia*

*Wolbachia* can also cause feminization in non-insect arthropods. *Wolbachia*-induced feminization in the woodlouse *Armadillidium vulgare* (Isopoda; Armadillidiidae) has a long research history [30]. As known in several insect species, the *A. vulgare Wolbachia* appear to be quite sensitive to high temperatures. Very young *Wolbachia*-infected females of *A. vulgare* reared at 30 °C gradually acquire a male phenotype [31,32]. In addition to *Wolbachia*, a non-bacterial feminizing factor (f) can also force chromosomal males of *A. vulgare* to become phenotypic functional females. The f factor is suspected to be a genetic element derived from the *Wolbachia* genome that becomes inserted into the host nuclear genome [33]. Genes resisting the feminizing effects or the transmission of feminizing elements have been found in natural populations of *A. vulgare* [34,35], thus illustrating the conflict between the feminizing elements and the rest of the host genome.

Unlike insects, androgenic hormone, a sex hormone secreted from a specific male organ called the androgenic gland, profoundly affects male sexual differentiation in *A. vulgare* [36,37]. In this respect, sex determination in *A. vulgare* seems to be more labile compared with that in insects. It is considered that *Wolbachia* feminize genetic males by disrupting the secretion of androgenic hormone. Before the discovery of feminization in *Eurema* butterflies, *Wolbachia* was assumed to be incapable of inducing complete feminization in insects [31]. This assumption appeared reasonable considering the substantial differences in the sex-determining systems between insects and non-insects. The actual occurrence of *Wolbachia*-induced feminization in both insects and non-insects may imply the presence of some common mechanism of sex determination that is targeted by *Wolbachia*. Alternatively, the feminization found in insects and non-insects may have distinct underlying mechanisms and only be superficially similar.

### 5.4. Feminization of Mites and Wasps by *Cardinium* Bacteria

The false spider mite, *Brevipalpus phoenicis* (Acarina; Tenuipalpidae), along with two closely related species, *Brevipalpus obovatus* and *Brevipalpus californicus*, is known to reproduce by thelytokous parthenogenesis [38]. Surprisingly, *B. phoenicis* females have a haploid genome, which was confirmed by fluorescence microscopy and variations at nine microsatellite loci [39]. Treatment of adult females with tetracycline hydrochloride led to a drastic increase in the proportion of male offspring (from 5.6% to 50.6%), suggesting that haploid individuals were feminized by a bacterium [40]. This bacterium is now known to be a member of the genus *Cardinium* [41]. Haploid individuals of the other species, *B. obovatus* and *B. californicus*, were also found to be feminized by *Cardinium* [42,43]. A feminizing effect of *Cardinium* was also found in the wasp *Encarsia hispida* (Hymenoptera; Aphelinidae), in which thelytokous parthenogenesis is known [44] (see Section 7.1).

### 5.5. Feminization of Shrimps by Microsporidian Parasites

Sex determination of the amphipod crustacean *Gammarus duebeni* (Amphipoda; Gammaridae) is more complex compared with that of insects. Basically, males and females are determined by a balance of a polyfactorial system of allelic sex genes located on several pairs of chromosomes, but the photoperiod can profoundly affect the sex determination [30]. Moreover, based on detailed breeding experiments, cytoplasmic factors were considered to act as female determiners in *G. duebeni* [45]. Subsequently, it was found that several microsporidian parasites such as *Octosporea effeminans*, *Nosema granulosis* and *Dictyocoela duebenum* living in the host cytoplasm alter male *G. duebeni* to functional females [46,47,48,49,50,51,52]. Rodgers-Gray *et al.* [50] demonstrated that *Nosema* manipulate the sex differentiation of *G. duebeni* by preventing androgenic gland differentiation, androgenic gland hormone production and consequently male differentiation. This is in agreement with observations of *Wolbachia*-induced feminization in *A. vulgare*. Although taxonomically unrelated (eukaryotes and prokaryotes), these feminizers may manipulate their crustacean hosts through a common mechanism.

### 5.6. Feminization Induced by Non-Microbes

Feminization can also be caused by non-microbes. According to Vance [53], more than 10% of wild-caught adults of the mayfly *Baetis bicaudatus* (Ephemeroptera; Baetidae) are parasitized by nematode *Gasteromermis* sp. (Nematoda; Mermithidae). None of the parasitized individuals (n = 126) contained visible eggs, ovaries or testes. Among them, the external morphology of 82 individuals (65%) was indistinguishable from that of normal females. The remaining 44 parasitized individuals showed an array of intersexual morphologies. Moreover, measurement of the DNA contents by flow cytometry suggested that the parasitized individuals having intersexual morphologies (“parasitized intersexes”) were genetically male individuals while parasitized individuals visibly indistinguishable from normal females (“parasitized females”) were composed of both genetically female and genetically male individuals [53]. The behaviors of the mayfly are also changed by the nematode. Unparasitized males form swarms near the river and do not return to the water after they have emerged. Vance [53] found that all 418 swarming individuals were unparasitized males and that the parasitized individuals showed ovipositing behavior, which is never seen in unparasitized males. Laboratory studies demonstrated that parasitized individuals (both parasitized females and parasitized intersexes) became very agitated shortly after emergence as adults. Within 3–6 hours, all the parasitized mayflies had crawled into the water down the side of the rock. The nematodes could then be seen escaping through a puncture wound in the mayfly’s abdomen [53]. Therefore, the *Gasteromermis* nematode can horizontally transmit to a new host. The mayflies were killed by the emergence of the nematode. The feminization in this case seems to be an adaptive strategy for the *Gasteromermis* nematodes, which do not have a vertical route of transmission.

Although less conspicuous than the case of the mayfly, perturbation of the development of secondary sexual characters by endosymbionts or parasites has also been found in various insects [54,55], in which the adaptive role of feminization remains unclear.

### 5.7. Masculinization Induced by Viruses

In woodlice such as *Porcellilo dilatatus*, *Porcellio laevis* and *A. vulgare*, an intersexual trait in genetic females is known to be transmitted from both parents. Viruses are considered to be the causal agent of the masculinization in genetic females because male characters disappeared after heat treatment, male characters appeared after injection of a 0.22-μm-filtered tissue extract and intersexuality was correlated with the presence of cytoplasmic viral particles [56]. The adaptive significance of the masculinizing effect remains unknown.

## 6. Endosymbiont-Induced Male Killing: An Easily Evolved Trait?

Compared with feminization, male killing is more common in insects (Table 2). Moreover, male killers have been found in taxonomically diverse microorganisms (e.g., bacteria in the genus *Spiroplasma*, *Wolbachia*, *Rickettsia*, *Arsenophonus*; unicellular eukaryotes in the phylum Microsporidia; and RNA viruses). Therefore, male killing may be easy to evolve [57]. Among them, *Wolbachia* and *Spiroplasma* are prevalent male killers among insects. In bacteria-induced male killing, only males are typically killed during embryonic or early larval stages which is called “early” male killing. On the other hand, in male killing induced by microsporidia and RNA viruses, the males are killed during later development (typically during the last stage of larva) which is called “late” male killing [58,59].

### 6.1. Function of Dosage Compensation is Necessary for Male Killing

*Drosophila* have two X chromosomes in females and only one X chromosome in males. By hypertranscribing the X-linked genes in males, *Drosophila* dissolve the imbalance in the gene dosage of the X-linked genes, which is often called dosage compensation. This process requires the formation of the dosage compensation complex (DCC), which consists of MSL-1, MSL-2, MSL-3, MLE and MOF [60]. 

How do male killers discriminate between males and females? Using classic genetic experiments, Veneti *et al.* [61] tested the ability of *Spiroplasma* to kill *D. melanogaster* males carrying mutations in genes encoding the DCC. Interestingly, *Spiroplasma* failed to kill males lacking any of the five protein components (MSL-1, MSL-2, MSL-3, MLE and MOF) of the DCC. Therefore, although the direct mechanism of male killing is elusive, their study clearly showed that the presence of the functional DCC is necessary for *Spiroplasma* to cause male killing in *D. melanogaster*.

### 6.2. Male Killing by Lethal Effect of Feminization?

**Figure 3:**
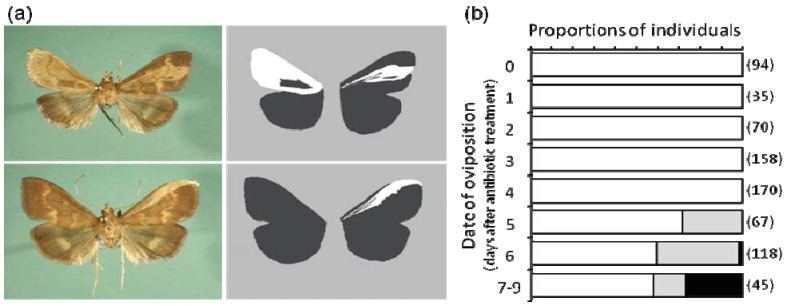
Effects of antibiotics on sexual phenotype of the moth *Ostrinia scapulalis* infected with *Wolbachia* [62]. (**a**) Intersexual individuals generated by tetracycline treatment. Black and white depict male-like color and female-like color, respectively. (**b**) The proportion of phenotypic females (white), intersexes (gray) and phenotypic males (black) among individuals whose mothers were treated with tetracycline hydrochloride prior to oviposition.

A mechanistic implication of male killing was obtained in our study on *Wolbachia*-induced male killing in the moth *Ostrinia scapulalis* (Lepidoptera; Crambidae), namely that males are killed by the feminizing effect of *Wolbachia*. Similar to other lepidopteran species, the sex chromosome constitution of *O. scapulalis* is ZZ in males and ZW in females. *Wolbachia*-infected females produce both ZZ and ZW offspring, but only ZZ offspring die during late embryonic and larval development. Interestingly, when adult females were fed with sucrose containing an antibiotic (tetracycline hydrochloride) prior to oviposition, they produced eggs that developed as intersexes as well as normal males and females [62,63,64] (Figure 3). The forewings of these intersexes showed a clear mosaic pattern of male-color and female-color sectors. Some of the intersexes had the bursa copulatrix, the female organ which accepts spermatophores from males during mating. Surprisingly, none of the tissues of the intersexes, including the bursa copulatrix, had a W chromosome, thus representing the pure male genotype (ZZ). Therefore, it is clear that the bursa copulatrix in these intersexes shows a female phenotype under a male genotype. Based on these findings, we concluded that *Wolbachia* has a feminizing effect on *Ostrinia* males but its full expression, which occurs in the natural condition, is lethal for genetic males. A reduction in the *Wolbachia* density during embryogenesis may result in partially feminized individuals (intersexes), probably due to the attenuated expression of the feminizing effect. The sex-determining gene *dsx* of these intersexes was shown to exhibit both male and female splicing patterns [65]. Reported very recently was conclusive evidence for the feminization as a mechanism of male killing in *Ostrinia*. In *Wolbachia*-infected *O. scapulalis*, female-specific splicing in *dsx* was observed in all the embryos including those with male genotype (ZZ) which were destined to die [66].

In addition, this case of male killing in *Ostrinia* showed another aspect when *Wolbachia* was completely eliminated. While other cases of male killing exhibit a normal 1:1 sex ratio after elimination of the male killers in their mothers, the elimination of *Wolbachia* from *Ostrinia* females by treatment with tetracycline hydrochloride during the whole larval stages resulted in the appearance of only males in the subsequent generation. This finding was erroneously interpreted as evidence for the *Wolbachia*-induced feminization [67,68]. However, later studies clearly showed that half of these offspring having female karyotype (ZW) die during late embryonic and larval development [63,64]. These individuals were recently shown to exhibit male-specific splicing in *dsx* [66], which implies that a female-determining factor, possibly located on W chromosome is degraded in *Ostrinia* and the *Wolbachia* substitutes the sex-determining role.

### 6.3. Hidden Male Killing

In some cases, male killing is masked or suppressed. *Wolbachia*-induced male killing was reported in the butterfly *Hypolimnas bolina* (Lepidoptera; Nymphalidae) [69,70]. In *H. bolina*, a strong female-biased sex ratio has been maintained for more than 100 years [71]. Surprisingly, in some populations, host resistance to male killing has recently spread and reached fixation [72,73,74]. In these populations, *Wolbachia* ceased to kill males but was not excluded from the host population. Hornett *et al.* [75] revealed that the same *Wolbachia* was inducing cytoplasmic incompatibility in the butterfly. This finding can explain the persistence of *Wolbachia* in the *H. bolina* population without inducing male killing.

In *Drosophila recens*, the sex ratios are not affected by naturally-occurring *Wolbachia*. This *Wolbachia* causes strong cytoplasmic incompatibility (see below) in *D. recens* and the infection frequency of *Wolbachia* in this species is 98%. On the other hand, none of the individuals are infected with *Wolbachia* in *Drosophila subquinaria*. Interestingly, introgression of the *D. recens Wolbachia* into *D. subquinaria* by hybridization and backcrossing resulted in the expression of male killing [76]. Furthermore, crossing experiments have demonstrated that the resistance to male killing is dominant, autosomal, multigenic and dependent on the zygotic, not maternal, genotype [76]. Similar to the case of *H. bolina*, male killing was masked by the fixation of resistant genes in *D. recens*. If there were no sister species, we would not have been able to reveal the potential for male killing in this insect.

The pea aphid *Acyrthosiphon pisum* is known to exhibit cyclical parthenogenesis. Previously, infection of *Spiroplasma* was only investigated under the asexual reproduction mode [77,78]. The effects of various endosymbionts in *A. pisum* under the sexual reproduction mode were recently analyzed and *Spiroplasma* was revealed to cause male killing [79]. The fact that *Spiroplasma* found in *A. pisum* was monophyletic with other male-killing *Spiroplasma* in ladybird beetles, butterflies and moths may indicate a common origin of the male-killing ability.

### 6.4. Is Timing of Male Killing Important?

Why do these microbes kill males? Killing only males during early development (early male killing) is considered advantageous for maternally transmitted microbes for three possible reasons that are not mutually exclusive [80,81]: (i) females can gain extra resources that were to be allocated to their brothers (resource reallocation); (ii) mating with sibling can be avoided (inbreeding avoidance); and (iii) microbes can be transmitted from dead (or dying) males to females by oral intake (horizontal transmission by cannibalism). On the other hand, resource reallocation cannot explain late male killing because males would consume nearly all the resources necessary for their own development before they are killed. Microsporidian parasites that cause late male killing in mosquitoes have intermediate hosts (copepods). Killing the mosquito larvae of large body size (late male killing) is considered to be an optimal strategy for the microsporidians because they can maximize their number and thereby increase their probability of transmission to copepod hosts [58,82]. 

Thus, the classification of male-killing phenotypes is based not only on the timing of the action, but also on the evolutionary strategies adopted by the symbionts and microbial agents responsible for the phenotypes (*i.e.*, bacteria versus eukaryotes and viruses). Therefore, it has been assumed that early and late male killing are fundamentally different phenomena whose underlying mechanisms are also entirely different [83,84]. In *Drosophila*, however, a *Spiroplasma* strain that normally cause early male killing was also found to induce late male killing depending on the maternal host age, in that male-specific mortality of larvae and pupae was more frequently observed in the offspring of young females [85]. Since the lowest *Spiroplasma* density and occasional male production were also associated with newly emerged females, we proposed a density-dependent hypothesis for the expression of the early and late male-killing phenotypes. Early male killing and late male killing could be regarded as alternative strategies adoptable by microbial reproductive manipulators [85].

The timing of male killing can be artificially changed in the *Wolbachia*-infected butterfly, *H. bolina*. Treatment of *Wolbachia*-infected adult females with tetracycline, a bacteriostatic antibiotic, produced a delay in the timing of male death. On the other hand, treatment of the surviving larvae with rifampicin, a bactericidal antibiotic, rescued the males. Based on these findings, it was hypothesized that *Wolbachia* possesses the ability to kill males through bacterial activity during larval development [86]. This phenomenon argues against the view that male killing is achieved by specifically targeting an early developmental process within the sex determination pathway. This is in line with the case of *Wolbachia*-induced feminization in the butterfly *E. mandarina*, wherein *Wolbachia* needs to be present during larval development for complete feminization [26].

## 7. Parthenogenesis Induction: Conversion of Genetic Males to Genetic Females or Feminization Following Diploidization?

Microbe-induced thelytokous parthenogenesis has been reported in haplodiploid arthropods, such as wasps, thrips and mites [19,87,88] (Table 2). On the other hand, there is no conclusive evidence for the microbe-induced parthenogenesis in diplodiploid organisms, although involvement of *Wolbachia* in thelytokous parthenogenesis in springtails and booklice is suspected based on the presence of *Wolbachia* [89]. In most of the known cases, microbe-induced parthenogenesis is achieved by altering the male genotype (haploid) to the female genotype (diploid), *i.e.*, restoration of diploidy. In this sense, the phenomenon can also be referred to as conversion of genetic males to genetic females [49]. At least in the wasp *E. hispida*, however, the restoration of diploidy is not sufficient for female development, and thus diploidization and feminization can be separate processes [44]. Frequently, certain species or populations are fixed for parthenogenesis-inducing microbes. In such cases, selection will not act on host genes involved in male and female sexuality (e.g., mating behaviors, sperm production, or sex pheromone production). Thus, mutations in such genes are expected to accumulate, resulting in eventual degeneration of sexuality and irreversible parthenogenesis. This process appears to be occurring in species of wasps [90,91,92] and thrips [88]. In contrast, sexual functions are not degenerated in *Trichogramma deion*, wherein parthenogenesis-inducing *Wolbachia* is not fixed and genetic exchange occurs between sexual and asexual individuals [93].

### 7.1. Mechanisms of Microbe-Induced Parthenogenesis

There are various mechanisms underlying microbe-induced parthenogenesis. Gamete duplication appears to be common in *Wolbachia*-infected hymenopteran wasps [93,94]. In wasps such as *Trichogramma* species and *Leptopilina clavipes*, meiosis is normal, but during the first mitotic division, the chromosomes fail to segregate in metaphase, resulting in diploidization of the nucleus [93,94]. In *Muscidifurax uniraptor*, on the other hand, meiosis and the first mitotic division are normal, but during the second mitotic division, diploid females are produced by the fusion of two cell nuclei [95]. Each offspring produced by gamete duplication is a homozygote at all loci and is not a genomic copy of its mother. On the other hand, the genotype of all offspring was indistinguishable from their mothers in *Rickettsia*-induced parthenogenesis occurring in *Neochrysocharis formosa* (Hymenoptera; Eulophidae) based on the polymorphisms in a microsatellite locus [96]. Moreover, by excluding the possibility of automictic parthenogenesis with central fusion, which has been observed in some ants [97,98], and using cytogenetic observations, Adachi-Hagimori *et al.* [96] concluded that apomictic parthenogenesis is the underlying mechanism wherein eggs do not undergo meiosis. Similarly, in the *Wolbachia*-induced parthenogenesis in the mite *Bryobia praetiosa*, the genotypes of the mothers and offspring are indistinguishable based on the polymorphisms in three microsatellite loci [40]. Therefore, apomictic parthenogenesis is the likely mechanism for this phenomenon as Weeks *et al.* [40] assumed, but another possibility of automictic parthenogenesis with central fusion cannot be completely excluded owing to the lack of cytogenetic observations.

It has been naturally assumed that diploidization induced by microbes automatically leads to female development. However, a recent finding indicates that, in the wasp *E. hispida*, *Cardinium*-induced parthenogenesis induction does not occur by diploidization alone. Feeding antibiotics to infected adult *E. hispida* females resulted in uninfected male offspring. By karyotype observations and flow cytometry analyses, Giorgini *et al.* [44] demonstrated that these males were diploid. This finding indicates that at least in *E. hispida*, diploidy restoration is necessary but not sufficient for female development. Thus, *Cardinium* is required to feminize diploid male embryos [44]. In this sense, this example should also be added to the list of microbe-induced feminization. As Giorgini *et al.* [44] argues, it might be necessary to consider the possibility that the mechanism of *Wolbachia*-induced parthenogenesis is also comprised of two separate steps, *i.e.*, diploidization and feminization.

## 8. Other Phenotypes

Here we briefly overview the other conspicuous effects caused by the above endosymbionts (Table S1).

### 8.1. Cytoplasmic Incompatibility

Cytoplasmic incompatibility is the most common phenotype of *Wolbachia* and *Cardinium* infection (Table S1). In diploid organisms, cytoplasmic incompatibility is an embryonic lethality that results in sperm and eggs having different cytoplasmic contents [17,99,100]. The effect arises from changes in the gamete cells caused by cytoplasmic (intracellular) parasites like *Wolbachia* and *Cardinium*, which infect a wide range of insect species. Cytoplasmic incompatibility occurs when a *Wolbachia*-infected male mates with a female that is either uninfected (unidirectional cytoplasmic incompatibility) or infected by another *Wolbachia* strain (bidirectional cytoplasmic incompatibility). Any other combinations of crosses are compatible. An infected female is compatible with an uninfected male or any infected male of the same *Wolbachia* strain. On the other hand, an uninfected female is only compatible with an uninfected male. 

Cytoplasmic incompatibility in haplodiploid hosts may lead to haploid (male) offspring. Cytoplasmic incompatibility produces distinct phenotypes in three closely related haplodiploid hymenopteran species of the genus *Nasonia*, namely mortality in *Nasonia longicornis* and *Nasonia giraulti*, and conversion to male development in *Nasonia vitripennis* [101].

### 8.2. Beneficial Effects of *Wolbachia* and *Spiroplasma* on Hosts

In addition to arthropods, some nematodes are also hosts of *Wolbachia* [102]. In filarial nematodes, *Wolbachia* does not seem to exhibit selfish behaviors like reproductive manipulations. Instead, *Wolbachia* is necessary for the host nematodes. Elimination of *Wolbachia* from filarial nematodes generally results in either death or sterility of the nematodes [103]. Consequently, current strategies for the control of filarial nematode diseases include elimination of *Wolbachia* via treatment with the antibiotic doxycycline rather than far more toxic anti-nematode medications [102].

In the bedbug *Cimex lectularius* (Hemiptera; Cimicidae), *Wolbachia* appears to be an obligate nutritional mutualist. *Wolbachia* is specifically localized in the bacteriomes, a specialized organ for endosymbiotic bacteria, and vertically transmitted via the somatic stem cell niche of germalia to oocytes. The transmitted *Wolbachia* infects the incipient symbiotic organ at an early stage of embryogenesis. Elimination of *Wolbachia* results in retarded growth and sterility of the host insect. However, these deficiencies are rescued by oral supplementation of B vitamins. These findings suggest that *Wolbachia* provides essential nutrition for this host [104].

In the parasitic wasp *Asobara tabida* (Hymenoptera; Braconidae), a strain of *Wolbachia* is necessary for oogenesis [105]. It is also known that *Wolbachia* rescues the oogenesis defect in a *Sex-lethal* mutant of *D. melanogaster* [106].

Increasing attention has been paid to the recent findings that *Wolbachia* can protect *Drosophila* against pathogenic RNA viruses such as *Drosophila* C virus, Cricket Paralysis virus, Flock House virus, Nora virus [107,108,109] and West Nile virus [110], as well as the fungus *Beauveria bassiana* [111]. Interestingly, in mosquitoes, the presence of transinfected *Wolbachia* interferes with a wider range of pathogens including nematodes and bacteria [112], Dengue virus and Chikungunya virus [113,114], as well as the avian and rodent malaria parasites *Plasmodium gallinaceum* [114] and *Plasmodium berghei* [115]. Biological control of mosquito-borne diseases is at the testing stage [116,117,118].

*Spiroplasma* also has similar effects on its hosts. *Drosophila neotestacea*, a fly that feeds on mushrooms, suffers complete loss of fecundity when parasitized by the nematode *Howardula aoronymphium* (Allantonematidae; Tylenchida). However, flies infected with *Spiroplasma* have near normal fecundity when parasitized by the nematode [119].

## 9. Transition of the Biological Systems of Arthropods by Sex-Associated Microbes

Despite the diversity of the sex-determining systems of arthropods and the diversity of microbial agents, the types of reproductive manipulation seem to be limited. For example, male killing occurs in male heterogametic insects, female heterogametic insects and haplodiploid insects. Moreover, the causal agents of male killing include a wide variety of bacteria as well as some eukaryotic organisms. Similarly, feminization occurs in arthropods having a ZW-ZZ system (butterflies, woodlice and shrimps), XX-X0 system (leafhoppers) and haplodiploid system (mayflies), and the causal agents of feminization also include bacteria and eukaryotes. Considering the stability of vertical transmission, these endosymbiotic microbes can be assumed to be sex-determining elements in the cytoplasm that work together with other sex-determining genes in the nucleus. It can also be assumed that the effects of these cytoplasmic sex-determining elements are relatively well conserved among arthropods.

Moreover, the presence of resistance genes against male killers and feminizers has been documented in some arthropods [73,76,120]. These resistance genes against microbial effects might be recognized as sex-determining genes. Although speculative, some of the sex-determining genes, such as *Sex-lethal*, *intersex*, *daughterless* and *sisterless* in *D. melanogaster*, could originally have arisen as resistance genes against the effects of the microbes and become fixed within the species. The diversity and complexity of the sex-determining systems of insects might have been generated by an arms race involving repeated invasion and/or mutation of various microbes and other genetic elements, which may not necessarily exist at the present time [2].

**Figure 4:**
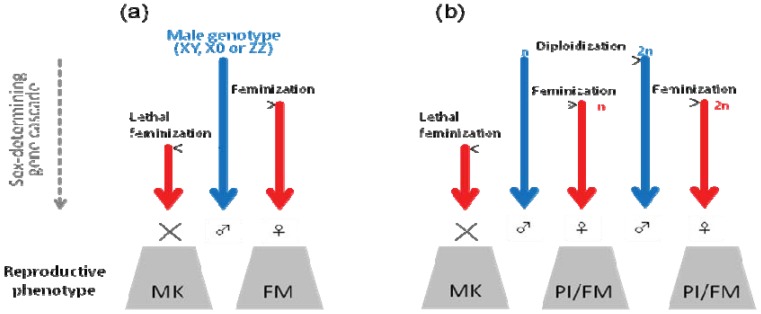
A parsimonious explanation of male killing (MK), feminization (FM) and parthenogenesis induction (PI). Here, MK, FM and PI are all assumed to be associated with the sex-determining process. The hypothetical flows of the male-determining and female-determining information are shown with blue and red arrows, respectively. Horizontal arrows indicate manipulation by endosymbionts. Note that this explanation is a hypothesis and may not necessarily reflect the actual mechanisms.

Currently, almost nothing is known about the molecular mechanisms of these acquired sex-determining systems. Regardless of the actual mechanisms, however, we can hypothesize a possible link between the microbial effects and the diversity of arthropod sex determination. As shown in Figure 4, one can place the possible effects of endosymbionts in the informational flow of sex-determining signals. To cause feminization, for example, endosymbionts need to simultaneously affect the sex determination in the germline, somatic line and central nervous system, each of which is considered to depend on a distinct expression cascade consisting of different genes. To achieve this, the endosymbionts may interfere with certain elements in the upstream of the sex-determining gene cascade that play pivotal roles, like *Sxl* in *Drosophila*, in sex determination in different lineages [4,121]. On the other hand, at least in *Ostrinia* moths, male killing may involve interactions with downstream genes, which may result in simultaneous expression of sexually antagonistic, male-specific and female-specific, genes and lead to lethality [63,65]. Although it was previously assumed that diploidization is the only effect of the parthenogenesis inducers in haplodiploid insects, recent findings in the *E. hispida* wasps imply that we need to take into account the possibility that endosymbionts may cause two distinct effects in order, *i.e.*, diploidization and feminization, the latter of which can be integrated into the feminization phenomena found in diploid insects. It is tempting to consider that, although endosymbionts work very simply (e.g., by only producing some proteins), the diversity and complexity of the host biology generate the diverse effects on the phenotype. A straightforward and frequently-used approach to elucidate the mechanisms of microbial effects on their hosts would be to compare the gene expression profiles between infected and uninfected insects. However, despite various attempts to compare the gene expression profiles [122,123,124,125], the mechanisms of reproductive manipulation still remain unclear. We consider that the mechanisms of microbe-induced reproductive manipulation will be clarified by a deeper understanding of insect development and physiology. Moreover, genomic data of endosymbionts and their hosts together with sophisticated mutagenesis techniques may allow reverse genetics approaches to elucidate the precise mechanism of reproductive manipulations.

## 10. Population-Level Effects of Endosymbiotic Microbes on Their Hosts

The spread and fixation of male killers and feminizers should lead to the absence of males and the extinction of the sexually reproducing hosts [126]. Moreover, a strongly-biased population sex ratio leads to a drastic reduction in the effective population size, which may inevitably cause inbreeding. Furthermore, inbreeding may cause a reduction in fecundity, which may increase the probability of extinction. In fact, a decrease in heterozygosity, an indication of inbreeding, was shown to increase the risk of extinction of island populations of the butterfly *Melitaea cinxia* [127]. However, endosymbiont-driven extinction has not been empirically demonstrated thus far.

Instead, in some butterflies, a *Wolbachia*-induced extreme bias in the population sex ratio was shown to be maintained for substantially long periods of time [71,128]. Moreover, resistance genes against male killers and feminizers have been documented [34,35]. Is this a usual reaction of hosts against the invasion of male killers and feminizers? We should be aware, however, that it is practically difficult to show the occurrence of population extinction caused by the spread and fixation of male killers and feminizers. Under the evolutionary timescale, the time required for sex-ratio distorters to spread among the host population can be too short for resistance genes to evolve. Owing to the short timeframe, the probability that we can observe a gradual decline in a population size is extremely low, even if endosymbiont-driven extinctions occur repeatedly in natural populations.

## 11. Conclusions

The accumulating data demonstrate that a variety of sexual aberrations in arthropods is imposed by endosymbionts (e.g., bacteria, protists, nematodes, insects and viruses). Although reducing the diverse phenomena to simple fundamental mechanisms may be dangerous and is not completely possible at present, some of the sexual aberrations can be explained by the interference with the sex-determining systems: for instance, male killing can be induced by the lethal effect of *Wolbachia*-induced feminization in a moth; parthenogenesis can be induced by *Cardinium* that feminizes haploid males in a mite; and diploid males are transformed by *Cardinium* into parthenogenetic females in a wasp. Importantly, these endosymbionts may profoundly affect host behavior, ecology and population structure. At present, the molecular mechanisms of any of the sexual aberrations are largely unknown. The deeper understanding of the arthropod physiology and development as well as arthropod and microbial genomes may allow us to elucidate the mechanism of the sexual aberrations, which will benefit both applied sciences (e.g., management of agricultural and medical pests and mass rearing of beneficial organisms) and other areas of biological sciences.

## Supplementary Materilas

**Table S1:** Other phenotypes induced by *Wolbachia*, *Spiroplasma* and *Cardinium*.

Phenotype	Endosymbiont	Class	Order	Species	Reference
CI	*Wolbachia*	Insecta	Coleoptera	*Callosobruchus chinensis*	[195]
CI	*Wolbachia*	Insecta	Coleoptera	*Chelymorpha alternans*	[196]
CI	*Wolbachia*	Insecta	Coleoptera	*D. virgifera*	[197]
CI	*Wolbachia*	Insecta	Coleoptera	*D. virgifera virgifera*	[197]
CI	*Wolbachia*	Insecta	Coleoptera	*D. virgifera zeae*	[197]
CI	*Wolbachia*	Insecta	Coleoptera	*Hypera postica*	[198,199,200]
CI	*Wolbachia*	Insecta	Coleoptera	*Tribolium confusum*	[201,202]
CI	*Wolbachia*	Insecta	Diptera	*Aedes albopictus*	[200]
CI	*Wolbachia*	Insecta	Diptera	*A. cooki *	[209,204]
CI	*Wolbachia*	Insecta	Diptera	*A. kesseli*	[205,206]
CI	*Wolbachia*	Insecta	Diptera	*A. malayensis*	[207,208]
CI	*Wolbachia*	Insecta	Diptera	*A. polynesiensis*	[207,208]
CI	*Wolbachia*	Insecta	Diptera	*A. pseudoscutellaris*	[209,210]
CI	*Wolbachia*	Insecta	Diptera	*A. s. scutellaris*	[209,211]
CI	*Wolbachia*	Insecta	Diptera	*Armigeres subalbatus*	[212]
CI	*Wolbachia*	Insecta	Diptera	*Culex pipiens*	[213,214,215]
CI	*Wolbachia*	Insecta	Diptera	*C. pipiens quinquefasciatus*	[216,217]
CI	*Wolbachia*	Insecta	Diptera	*Drosophila auraria*	[218]
CI	*Wolbachia*	Insecta	Diptera	*D. melanogaster*	[219,220]
CI	*Wolbachia*	Insecta	Diptera	*D. recens*	[221]
CI	*Wolbachia*	Insecta	Diptera	*D. sechellia*	[222]
CI	*Wolbachia*	Insecta	Diptera	*D. simulans*	[219,224]
CI	*Wolbachia*	Insecta	Diptera	*Liriomyza trifolii*	[224]
CI	*Wolbachia*	Insecta	Diptera	*Phlebotomus papatasi*	[225]
CI	*Wolbachia*	Insecta	Diptera	*Rhagoletis cerasi*	[226,227]
CI	*Wolbachia*	Insecta	Hymenoptera	*Aphytis melinus DeBach *	[228]
CI	*Wolbachia*	Insecta	Hymenoptera	*Asobara tabida*	[229]
CI	*Wolbachia*	Insecta	Hymenoptera	*Cotesia sesamiae*	[230]
CI	*Wolbachia*	Insecta	Hymenoptera	*Encarsia inaron*	[231]
CI	*Wolbachia*	Insecta	Hemiptera	*Laodelphax striatellus*	[232,233,234]
CI	*Wolbachia*	Insecta	Hemiptera	*Leptopilina heterotoma*	[235,236,237]
CI	*Wolbachia*	Insecta	Hemiptera	*Macrolophus pygmaeus*	[238]
CI	*Wolbachia*	Insecta	Hemiptera	*Nasonia giraulti*	[239,240]
CI	*Wolbachia*	Insecta	Hemiptera	*N. longicornis*	[240,241]
CI	*Wolbachia*	Insecta	Hemiptera	*N. vitripennis*	[240,242]
CI	*Wolbachia*	Insecta	Hemiptera	*Orius strigicollis*	[243]
CI	*Wolbachia*	Insecta	Lepidoptera	*Colias erate poliographus*	[244]
CI	*Wolbachia*	Insecta	Lepidoptera	*Ephestia cautella*	[245]
CI	*Wolbachia*	Insecta	Lepidoptera	*E. kuehniella*	[246]
CI	*Wolbachia*	Insecta	Lepidoptera	*Eurema hecabe*	[247]
CI	*Wolbachia*	Insecta	Lepidoptera	*E. madarina*	[248,249]
CI	*Wolbachia*	Insecta	Orthoptera	*Gryllus assimilis*	[197]
CI	*Wolbachia*	Insecta	Orthoptera	*G. integer*	[197]
CI	*Wolbachia*	Insecta	Orthoptera	*G. ovisopis*	[197]
CI	*Wolbachia*	Insecta	Orthoptera	*G. pennsylvanicus*	[197]
CI	*Wolbachia*	Insecta	Orthoptera	*G. rubens*	[197]
CI	*Wolbachia*	Insecta	Orthoptera	*Teleogryllus taiwanemma *	[249]
CI	*Wolbachia*	Malacostraca	Isopoda	*Cylisticus convexus*	[250]
CI	*Wolbachia*	Malacostraca	Isopoda	*Porcellio dilatatus*	[233,251]
CI	*Wolbachia*	Arachnida	Trombidiformes	*Oligonychus gotohi*	[252]
CI	*Wolbachia*	Arachnida	Trombidiformes	*Panonychus mori*	[252,253]
CI	*Wolbachia*	Arachnida	Trombidiformes	*Tetranychus urticae*	[253,254]
CI	*Cardinium*	Insecta	Hymenoptera	*Encarsia pergandiella*	[257]
CI	*Cardinium*	Arachnida	Mesostigmata	*Dermanyssus gallinae*	[258]
CI	*Cardinium*	Arachnida	Trombidiformes	*Bryobia sarothamni*	[289]
CI	*Cardinium*	Arachnida	Trombidiformes	*Eotetranychus suginamensis*	[256]
CI	*Cardinium*	Arachnida	Trombidiformes	*Tetranychus cinnabarinus*	[260]
RP ^a^	*Wolbachia*	Insecta	Diptera	*Anopheles gambiae*	[261]
RP ^b^	*Wolbachia*	Insecta	Diptera	*Aedes aegypti*	[262,263,264]
RP ^c^	*Wolbachia*	Insecta	Diptera	*Culex quinquefasciatus*	[265]
RP ^d^	*Wolbachia*	Insecta	Diptera	*Drosophila simulans*	[266,267]
RP ^e^	*Wolbachia*	Insecta	Diptera	*D. melanogaster*	[265,268,269,270]
RP^ f^	*Spiroplasma*	Insecta	Diptera	*D. hydei*	[271]
RP ^g^	*Spiroplasma*	Insecta	Diptera	*D. neotestacea*	[272]
M	*Wolbachia*	Insecta	Hemiptera	*Cimex lectularius*	[273]
M	*Wolbachia*	Secernentea	Spirurida	*Brugia malayi *	[274-271]
M	*Wolbachia*	Secernentea	Spirurida	*B. pahangi*	[277]
M	*Wolbachia*	Secernentea	Spirurida	*Dirofilaria immitis*	[277,278]
M	*Wolbachia*	Secernentea	Spirurida	*Litomosoides sigmodontis*	[279,280]
M	*Wolbachia*	Secernentea	Spirurida	*Onchocerca gutturosa *	[281]
M	*Wolbachia*	Secernentea	Spirurida	*O. lienalis*	[281]
M	*Wolbachia*	Secernentea	Spirurida	*O. ochengi*	[282]
M	*Wolbachia*	Secernentea	Spirurida	*O. volvulus*	[283]
M	*Wolbachia*	Secernentea	Spirurida	*Wuchereria bancroft*	[284]
O	*Wolbachia*	Insecta	Hymenoptera	*Asobara tabida*	[236]
O	*Wolbachia*	Insecta	Coleoptera	*Coccotrypes dactyliperda *	[285]
O	*Wolbachia*	Insecta	Coleoptera	*Otiorhynchus sulcatus*	[286]
O	*Wolbachia*	Insecta	Collembola	*Folsomia candida*	[287]
O	*Wolbachia*	Insecta	Diptera	*Drosophila melanogaster *(*Sxl*)	[288]

CI, cytoplasmic incompatibility; RP, resistance to pathogen; M mutualism; O; oogenesis.

^a^ Confer resistance to *Plasmodium berghei * and *P. falciparum*

^b^ Confer resistance to *Plasmodium gallinaceum, *Dengue, Chikungunya and *Brugia pahangi*

^c^ Confer resistance to West Nile virus

^d^ Confer resistance to *Drosophila *C virus, Flock House virus, Dengue

^e ^Confer resistance to *Drosophila* C virus, Nora virus, Flock House virus, West Nile virus and *Blauveria bassiana*

^f^ Confer resistance to the parasitic wasp *Leptopilina heterotoma*

^g^ Confer resistance to the nematode *Howardula aoronymphium*
